# Supplementary material for: Tead1a Initiates Transcriptional Priming Through the TEAD1a/YAP‐Notch1‐Spi1/Cebpα Axis to Promote Neutrophil Fate
Source: Adv Sci (Weinh). 2025 Aug 28;12(41):e05441. doi: 10.1002/advs.202505441 (PMC12591127; doi:10.1002/advs.202505441)
Supplement: Supplementary file 4 — Supplementary Dataset [file ADVS-12-e05441-s004.zip › 3-2.zip!/3-2/02-2.cluster/cluster3/Enrich/ego_kegg.pdf]

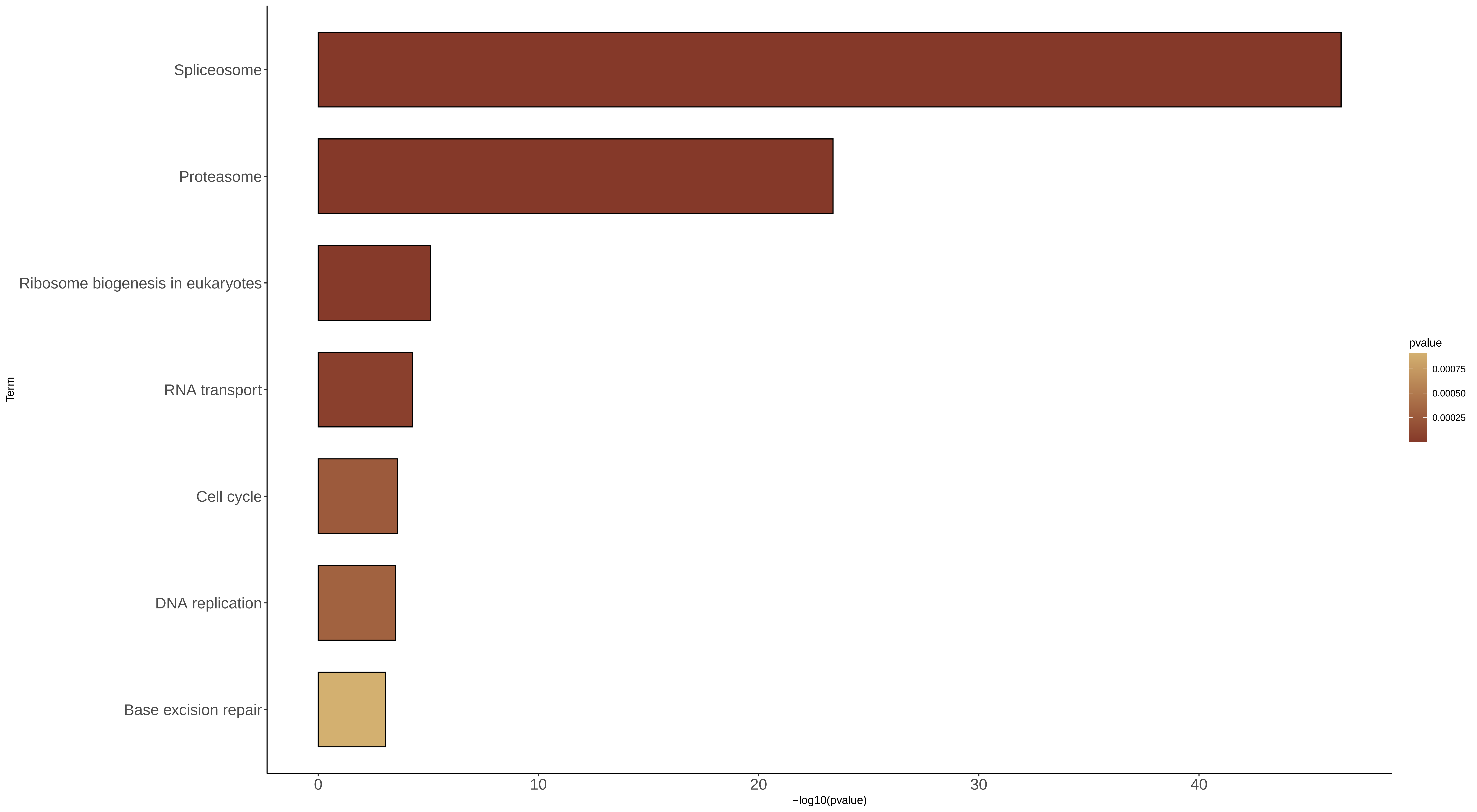
Spliceosome
Proteasome
Ribosome biogenesis in eukaryotes
RNA transport
Cell cycle
DNA replication
Base excision repair
Term
0
10
20
30
40
−log10(pvalue)
pvalue
0.00075
0.00050
0.00025
